# Targeted NGS Yields Plentiful Ultra-Rare Variants in Inborn Errors of Immunity Patients

**DOI:** 10.3390/genes12091299

**Published:** 2021-08-24

**Authors:** Alice Grossi, Maurizio Miano, Marina Lanciotti, Francesca Fioredda, Daniela Guardo, Elena Palmisani, Paola Terranova, Giuseppe Santamaria, Francesco Caroli, Roberta Caorsi, Stefano Volpi, Marco Gattorno, Carlo Dufour, Isabella Ceccherini

**Affiliations:** 1 UOSD Laboratory of Genetics and Genomics of Rare Diseases, IRCCS Istituto Giannina Gaslini, 16148 Genoa, Italy; alice_grossi@libero.it (A.G.); giuseppesantamaria@gaslini.org (G.S.); genseq@unige.it (F.C.); 2 Hematology-Oncology Stem Cell Transplantation Pole, IRCCS Istituto Giannina Gaslini, 16148 Genoa, Italy; mauriziomiano@gaslini.org (M.M.); marinalanciotti@gaslini.org (M.L.); francescafioredda@gaslini.org (F.F.); danielaguardo@gaslini.org (D.G.); elenapalmisani@gaslini.org (E.P.); paolaterranova@gaslini.org (P.T.); carlodufour@gaslini.org (C.D.); 3 Center for Autoinflammatory Diseases and Immune-Deficiencies, IRCCS Istituto Giannina Gaslini, 16148 Genoa, Italy; robertacaorsi@gaslini.org (R.C.); Stefanovolpi@gaslini.org (S.V.); marcogattorno@gaslini.org (M.G.)

**Keywords:** bone marrow failure, autoinflammation, lymphoproliferation, next-generation sequencing (NGS), NGS-based gene panels, genotype-phenotype correlation

## Abstract

Inborn errors of immunity (IEI) include a large group of inherited diseases sharing either poor, dysregulated, or absent and/or acquired function in one or more components of the immune system. Next-generation sequencing (NGS) has driven a rapid increase in the recognition of such defects, though the wide heterogeneity of genetically diverse but phenotypically overlapping diseases has often prevented the molecular characterization of the most complex patients. Two hundred and seventy-two patients were submitted to three successive NGS-based gene panels composed of 58, 146, and 312 genes. Along with pathogenic and likely pathogenic causative gene variants, accounting for the corresponding disorders (37/272 patients, 13.6%), a number of either rare (probably) damaging variants in genes unrelated to patients’ phenotype, variants of unknown significance (VUS) in genes consistent with their clinics, or apparently inconsistent benign, likely benign, or VUS variants were also detected. Finally, a remarkable amount of yet unreported variants of unknown significance were also found, often recurring in our dataset. The NGS approach demonstrated an expected IEI diagnostic rate. However, defining the appropriate list of genes for these panels may not be straightforward, and the application of unbiased approaches should be taken into consideration, especially when patients show atypical clinical pictures.

## 1. Introduction

Inborn errors of immunity (IEI) include a large heterogeneous group of inherited diseases sharing either poor, dysregulated, or absent and/or acquired function in one or more components of the immune system. More than 400 different monogenic immune disorders and corresponding genes have been identified to date, and many new others are continuously being recognized [1]. Some of these disorders are chronic and severe, and timely diagnosis can allow identifying targeted drug treatment(s) and/or the suitable conditioning regimen when bone marrow transplantation is needed [2,3,4]. With the exception of IgA deficiency (1/300–1/500), IEI are more frequent than previously believed, with an estimated overall prevalence of 1 in 1200 live births [5], and can be classified based on whether the affected component belongs to either the adaptive or innate immune system [6]. A distinction is also made with secondary immune deficiencies resulting from other causes such as viral or bacterial infections, malnutrition, treatments that induce immunosuppression, or immunoglobulin loss [5,7].

In recent years, novel monogenic disorders characterized by clinical signs of immune dysregulation have been identified in the group of IEI and defined as primary immuno-regulatory disorders (PIRDS) [8]. The majority of them belong to the clinical spectrum of autoimmune lymphoproliferative syndrome (ALPS) or common variable immunodeficiency (CVID), showing most of the clinical signs and symptoms (such as autoimmunity and chronic benign lymphoproliferation) without completely fulfilling the diagnostic criteria [9,10], and for this reason, they are also often named ALPS-like or CVID-like disorders. 

The diagnostic approach to IEI has been dominated, thus far, by time-consuming phenotypic and functional characterization [10,11,12]. More recently, molecular genetic testing has emerged as an essential tool often providing a conclusive diagnosis also in atypical cases, assisting in genetic counseling, prenatal diagnosis, carrier identification, and precision therapeutics. Genetic testing has also allowed drawing genotype-phenotype correlations, often lacking due to reduced penetrance and variable expressivity, to disclose the wide phenotypic heterogeneity due to allelic series, and to reveal many genetically diverse but phenotypically overlapping diseases [13,14,15]. The advent of next-generation sequencing (NGS) has driven the rapid increase in recognizable IEI, also leading to the discovery of new genes implicated in well-defined biological pathways [16,17,18,19,20,21,22,23,24]. This enabled the characterization of new disorders, and the attribution of new clinical phenotypes to underlying genetic variants of already known diseases, thus narrowing the gap between hematology, immunology, and rheumatology [25]. Indeed, the multifaceted phenotype of IEI, including infections, autoimmunity, autoinflammation, allergy, and/or malignancy, is challenging, with many implications for effective diagnostic work-up, relevant treatment, and correct follow-up [21,26,27].

Whole-exome sequencing (WES) and whole-genome sequencing (WGS) have allowed detecting around 150 new variants (nearly 40% of all currently known mutations) thus far [22]. However, gene panels are faster and cheaper than unbiased sequencing and provide a much more limited number of variants to interpret, thus raising fewer interpretation problems than WES/WGS. Indeed, cost, accessibility, and interpretation are major challenges to using genetic testing for the evaluation of IEI [28]. 

After testing three different NGS-based gene panels in patients affected with IEI, we report the variants detected, their frequency and recurrence, and the associated diseases, thus contributing to disclosing the wide genetic variability of lymphoproliferation, autoimmune/idiopathic cytopenia, autoinflammation, and bone marrow failure.

## 2. Materials and Methods 

### 2.1. Patient Recruitment

Patients referred to both the Hematology Unit and Center for Autoinflammatory Diseases and Immunedeficiencies of the Istituto Giannina Gaslini were selected either retrospectively and still undiagnosed or prospectively for new referrals, independently of age, sex, and ethnicity. Inclusion criteria were considered the presence of at least one of the following: (i) single-/multilineage bone marrow failure (BMF), (ii) autoimmune hemolytic anemia, (iii) neutropenia, (iv) chronic ITP, (v) multilineage autoimmune cytopenia, (vi) benign chronic lymphoproliferation lasting > 6 months, (vii) clinical/biochemical signs of autoimmunity or autoinflammation requiring treatments. All adult subjects provided written informed consent to participate in this study, while parental consent was obtained for children, as approved by the Istituto Gaslini Ethical Committee.

### 2.2. Study Design 

DNA was isolated from peripheral blood samples of patients, and parents when available, and extracted by using QIAamp DNA Blood Midi kit (Qiagen, Germantown, MD, USA). The quality and quantity of DNA thus obtained were determined by a Nanodrop.

To genetically define patients with either unclassified cytopenias (either central or peripheral) or autoinflammation on the background of an underlying immune dysregulation, from December 2015 to December 2019, we designed three consecutive gene panels (Supplementary Table S1). These were used on three successive and non-overlapping temporal periods. Overall, genes were selected according to different purposes, based on the 2017 report of the International Union of Immunological Societies (IUIS) [19] as well as the most up-to-date literature reports [29]. Chronologically, the first panel (Emato-Immunological Panel) included 146 genes related to marrow failure, cytopenia, and immune dysregulation. The second panel (Comprehensive Immune Dysregulation Panel) contained 312 genes responsible for a wide IEI spectrum, mostly not included in the first one. Based on the results achieved with these first two panels, the third one (Hematological Routine Panel) was a synthesis panel with 58 genes, sharing a core set of 52 genes with the two above panels, originally aimed to become a routine diagnostic tool for a majority of newly identified cases presenting the above features.

### 2.3. Library Design and Sequencing, Bioinformatic Analysis, and Sanger Validation

Patients were subjected to massive parallel sequencing and successive bioinformatics analyses as described in the Supplementary Information and Supplementary Tables S2 and S3.

## 3. Results

A total of 272 unrelated patients (142 male and 130 female, mean age 15.5 years), already assessed through conventional clinical evaluations and found to be affected with ALPS, CVID, and other PIRDS (*n* = 164), bone marrow failure (*n* = 40), idiopathic neutropenia (*n* = 35), systemic autoinflammatory disease (SAID) (*n* = 12), immune deficiencies other than PIRDS (*n* = 11), autoimmune hemolytic anemia (AIHA) (*n* = 6), hemophagocytic lympho-histiocytosis (HLH) (*n* = 2), complement defect (*n* = 1), and hyper-eosinophilia (*n* = 1), were tested for possible variants of genes, selected as described above. A total of 68 of the 272 enrolled patients (25%) had undergone previous genetic studies, as reported for those shown in Table 1, Table 2, and Supplementary Table S4, together with the candidate gene(s) tested earlier [30,31]. In no case did these previous analyses identify the causal gene.

**Figure 1:**
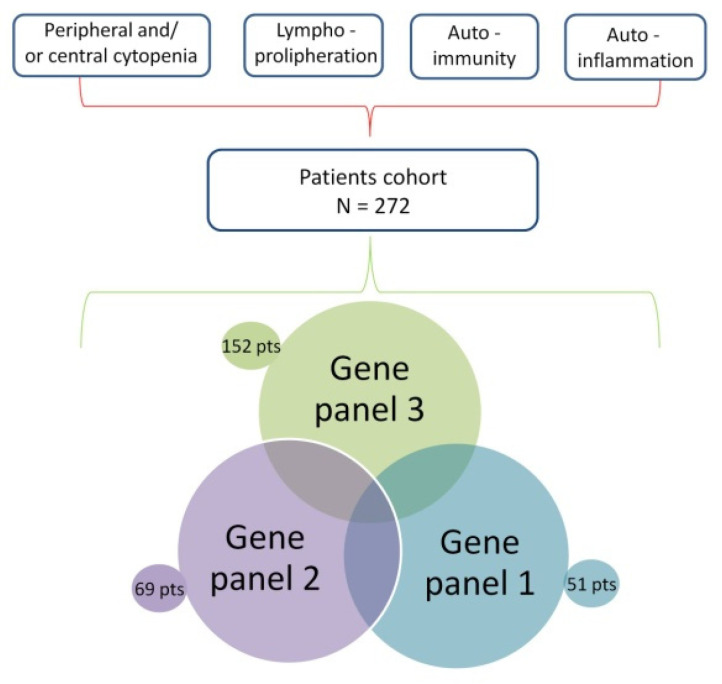
Breakdown of a total of 272 patients with unclassified cytopenias (either central or peripheral), immune dysregulation, autoimmunity, and autoinflammation that underwent genetic tests at the Gaslini Institute from 2015 to 2019, through the three consecutive overlapping gene panels shown at the bottom.

According to the experimental design reported in Figure 1, 51, 69, and 152 patients were tested using three consecutive panels including 146, 312, and 58 genes, respectively, one patient (ID38) having been analyzed in both panels 1 and 2 to increase the chances for genetic definition. The gene composition of the three panels is reported in Supplementary Table S1.

A total of 197 rare variants, representing 247 alleles, were detected across the three panels, validated through Sanger sequencing, and assessed for their potential effects. As summarized in Table 3, 47 of them are predicted to be pathogenic or likely pathogenic, 33 variants have a probable effect on the phenotype, though with an unknown significance, and the remaining 117 variants are very heterogeneous but expected to have no effect on the clinical phenotype. The total 197 variants are distributed across the 272 patients, with variable proportions of them carrying 0, 1, 2, and ≥3 variants, as reported in Table 3. Overall, 24 variants recur among the patients. 

### 3.1. Pathogenic or likely Pathogenic Variants Detected

The 47 variants predicted pathogenic or likely pathogenic, representing 57 alleles detected in 51 patients, are reported in Table 1. According to the classification criteria described in the “Supplementary Information”, only 42 out of these 57 variant alleles, carried by 37 patients, account for the associated phenotypes. These 37 patients, shown in the gray lines in Table 1, carry causative (pathogenic/likely pathogenic) variants of genes, compatible, to a variable extent, with their clinics, showing a zygosity consistent with the inheritance mode of the disease, thus yielding a 37/272 (13.6%) diagnostic rate. A total of 12 of the 47 pathogenic or likely pathogenic variants (25.5%) are reported in neither the GnomAD v.3 database (https://gnomad.broadinstitute.org/ (accessed on 14 January 2021)) [32] nor the dbSNP, having been detected here for the first time. Finally, five variants, one variant, and one variant recur in two, three, and four patients, respectively, thus strengthening their role in the immune dysregulation of the corresponding patients. 

Some of the pathogenic or likely pathogenic variants found in patients showing both typical and atypical clinical manifestations already have functional evidence/suggestions; however, in other cases, the variant effect is still uncertain/not confirmed.

Patient ID32 showed three different causative variants. One rare *STAT3* variant was a somatic mosaicism, being present in the DNA extracted from peripheral blood but not in the DNA extracted from a different source. Two other rare variants affected the two different alleles of the *ADA2* gene: indeed, the parents turned out to carry one variant each (p.Leu188Pro for the mother, and p.Thr187Pro for the father). The *ADA2* deficiency (DADA2) in this patient and her sister, having the same *ADA2* genotype, manifested with ALPS-like symptoms, as already reported for some DADA2 cases [33,34,35].

Patient ID1176 carried two rare alleles of the *MVK* gene, being a compound heterozygote for c.503_508delTGAAGG (p.Leu168_Asp170delinsHis), transmitted from his mother, and c.1129G>A (p.Val377Ile), transmitted from his father. This genotype is consistent with an MKD diagnosis that was clinically complicated with an onset characterized by ALPS-like symptoms.

Patients ID15, ID39, ID94, and ID97 are homozygous for the deleterious *RAG1, LRBA, NCF1*, and *IL7R* gene variants, respectively, in three of which, except for ID97, the parents demonstrated being heterozygotes. These cases are consistent with the autosomal recessive inheritance of the corresponding diseases and with the clinical features of the patients [36,37]. 

Patient ID226 carried multiple pathogenic or likely pathogenic variants that may have contributed to the disease outcome. In fact, though the *ELANE* mutation alone, unreported in the GnomAD database thus far, can explain the cyclic neutropenia of this patient, we cannot exclude that *TNFRSF13B*, present with a variant showing no homozygotes in the same database, may also be involved in the clinical phenotype [38]. 

Patients ID80 and ID260 carried pathogenic variants in the X-linked *IKBKG* and the *CARD11* genes, respectively, showing a clinical overlap between IEI and bone marrow failure, in line with what was recently demonstrated [34]. The CARD11 variant is unreported thus far.

On the other hand, most of the 13 patients shown in the blank lines in Table 1 are heterozygous for variants that, though predicted with a causative effect, are responsible for recessively inherited phenotypes. These patients are therefore expected to be asymptomatic carriers for the respective diseases, with the immune dysregulation disorders they are affected by likely caused by variants of other genes untested here or by the presence of a null allele undetected in *trans* at the same locus. Indeed, among these latter cases, the *TNFRSF13B* p.Glu117Glyfs*35 variant, though predicted to be of an unknown significance, found in the ID100 patient (see Table 2 and next paragraph) might account for, either alone or with the heterozygous *AIRE* p.Glu517Ter variant (Table 1), her ALPS-like phenotype. 

Finally, patient ID10 carried a yet unreported pathogenic variant of the complement component 9 (*C9*), whose defects still have an undefined mode of inheritance [39], with no consistent symptoms.

Disorders that had a more accurate diagnosis were immunodeficiency (2/11, 18%), SAID (2/12, 16.7%), ALPS/ALPS-like (25/164, 15.2%), BMF (5/40, 12.5%), and undefined neutropenia (4/35, 11.4%), while no patient affected by HLH, AIHA, complement defect, and hyper-eosinophilia could be genetically assessed. A genetic confirmation of the clinical suspicion could not be achieved for 235/272 patients (86%) that have therefore remained unexplained, with either rare (probably) damaging variants in genes unrelated to their phenotype, variants of unknown significance in genes consistent with their clinics, or apparently inconsistent benign, likely benign, or VUS variants.

### 3.2. Variants of Unknown Significance with a Probable Effect on the Phenotype

Among these latter undiagnosed cases, the patients and corresponding variants reported in Table 2 deserve to be taken into account. This is the case of variants classified as having an unknown significance, as unreported thus far in association with corresponding diseases, being in fact very rare with pathogenicity scores predicting damaging effects. Indeed, these 33 variants were selected as potentially having a probable effect on patients’ phenotypes based on: (i) frequency in the general population (F < 0.005), (ii) in silico prediction of adverse functional effects, namely, CADD > 20 and/or DANN > 0.98, with at least one of the FATHMM, SIFT, and PROVEAN software packages predicting a damaging effect, and (iii) showing a single occurrence, with no other cases carrying the same variant.

The six patients shown in the gray lines in Table 2 are those carrying variants that affect genes whose defects are consistent with the corresponding patients’ clinics and with zygosity concordant with the inheritance mode of the disease, thus likely accounting for the associated phenotypes. The case of patient ID2 is particularly evocative in the light of the de novo occurrence of her *PRKCD* variant. Indeed, none of these variants of unknown significance have ever been found in homozygotes, except for the TERT variant p.Glu441del detected in the heterozygous state in patient ID203 and also in two homozygotes in the GnomAD v.3 database. For this reason, this variant is unlikely to have had an impact on the resulting patient’s phenotype, which may be sustained, instead, by a TNFRSF13B variant already reported in Table 1.

Assuming these additional five patients as solved cases, the diagnostic yield would increase to a further 5/272 (1.8%) rate. The overall success rate achieved in the present study, considering the sole patients carrying probably causative variants reported in Table 1 and Table 2, was therefore 42/272 (15.4%), with 9/51 (17.6%), 16/69 (23.2%), and 17/152 (11.2%) from the first to the third panels, respectively. Interestingly, given the 146, 312, and 58 genes in panels 1, 2, and 3, a correlation between the number of genes in each panel and the proportion of patients whose diagnosis could be confirmed can be proposed.

### 3.3. Wide Genetic Variability in Immune Dysregulation Disorders: Low-Impact Variants

The remaining 117 variants, representing 157 alleles, given 10 variants recurring twice, 2 variants recurring 3 times, 2 variants recurring 5 times, and 3 variants each recurring in 6, 7, and 8 unrelated patients, are reported in Supplementary Table S4. These were classified as benign, likely benign, or variants of unknown significance, the last classification presumably having a low impact on patients’ phenotypes due to either a frequency of >0.005 in the general population, in silico prediction of tolerant/neutral functional effects, with CADD < 20 and/or DANN < 0.98, or at least one of the FATHMM, SIFT, and PROVEAN software packages predicting no damaging effect.

Despite the improbable role of any of these 117 variants in the corresponding patients’ clinical phenotypes, we depicted those lines reporting variants that seem to have reasons to still be considered in gray. These total 21 variants, representing 51 alleles, mostly predicted with a benign effect (36/51, 70.6%) and, in some cases, already detected in homozygotes of the general population (see Supplementary Table S4). These variants show a high degree of recurrence, including from 2 to 8 patients carrying the same variant for a total of 39 alleles present in more than one patient (39/51, 76.4%). The p.His159Tyr *TNFRSF13C* variant, for instance, has an 8.2 × 10^−3^ frequency in the European non-Finnish population of the GnomAD v.3 database and has also been found in three homozygotes, but it is present in 8/272 (2.94 × 10^−2^) patients, that is, it is 3.6 times more frequent in our case set. Similarly, the p.Arg202His *TNFRSF13B* and the p.Pro501Leu *CASP10* variants, both undetected thus far in homozygosity, each present in 2/272 patients, and the p.Met309Ile *ADA2* variant present in 3/272 patients are nearly 7, 26, and 6 times more frequent in our dataset than in the general population, respectively. However, further analyses, carried out by using a more appropriate set of Italian healthy controls, are required before assessing the potential involvement of these rare recurring variants in the susceptibility of the corresponding diseases. Furthermore, although the frequency of the p.Ser312Cys PIK3CD variant appears to be comparable between the general population (0.0187) and our set of patients (0.0257), and 45 homozygotes are reported in the GnomAD database, molecular studies in vitro have shown an altered function [34]. In addition, some of the above 21 variants affect genes whose impact in immune dysregulation may need to be re-evaluated, such as *TNFRSF13B* [40], *RAG1* [41,42], *TNFRSF13C* [43], *CASP10* [44], and *PIK3CD* [34].

## 4. Discussion

Inborn errors of immunity (IEI) are clinically heterogeneous entities arising from defects in genes involved in immunity whose effects extend well beyond susceptibility to infection, including multiorgan autoimmunity, hematological diseases, or autoinflammatory conditions. Although IEI are considered Mendelian disorders, massive targeted sequencing of undiagnosed patients has not led to a significant improvement in the diagnostic yield, but rather to a growing discovery of new variants often presenting imperfect inheritance patterns and wide phenotypic heterogeneity, thus complicating the diagnostic assessment. A polygenic mode of inheritance has also been postulated in some cases [15].

This prompted us to develop three different temporarily consecutive next-generation sequencing (NGS)-based gene panels that were used to test patients presenting with complex and/or atypical phenotypes highly suggestive of IEI and yet undiagnosed after testing candidate genes by the traditional Sanger sequencing protocol [45]. Indeed, atypical presentations may be missed when focusing on given phenotypes, and, conversely, larger NGS-based gene panels can lead to identifying variants unseen before and/or in genes whose contribution to a given disease phenotype is not yet completely established [25]. 

Consistent with such expectations, the rare variants filtered at first turned out to be pathogenic or likely pathogenic, either associated with compatible phenotypes or, conversely, affecting genes unrelated to the patient’s disease. In addition, we could also detect variants of unknown significance, extremely rare with significant damaging scores, in genes consistent with the corresponding phenotypes, as well as heterozygous variants, predicted either as damaging or tolerated, in genes responsible for autosomal recessive traits. Finally, a vast multitude of rare benign, likely benign, or VUS variants, apparently unable to explain the underlying diseases, were also found.

The demand for NGS-based testing has grown rapidly due to its advantages compared to conventional genetic testing (higher mutation yields, more genes simultaneously tested, much larger patient sets under study) without, however, a corresponding increase in the rate of detection of causative variants, the yield of the most focused panels for different diseases varying in the literature from 15–25% to 40–50% [11,12,16,17,19,43,46]. Even with the use of clinical exome sequencing (CES), the diagnostic yield did not exceed 32% [23].

In agreement with statistics already reported in the literature [47], here, we obtained 37/272 (13.6%) patients for whom a genetic diagnostic confirmation could be achieved, given the detection of pathogenic and likely pathogenic variants consistent with the corresponding disorders and mode of inheritance. Though taking VUS variants into consideration for diagnostic purposes may be questionable, variant classification is subjected to change as new information emerges, making the prediction of variant effects deeply dynamic. For this reason, we also tentatively took into account variants of unknown significance in potentially causative genes, found in 6/272 patients, to obtain an overall rate of 43/272 (15.8%). The proportion of solved cases across the three gene panels adopted reflects a relationship with the gene panel extensions, consistent with the most severe historical cases having been tested in gene panel 1. Thus, if a careful selection of patients can lead to higher diagnostic rates even in smaller gene panels, larger panels have the advantage of allowing the detection of overlaps (i.e., variant co-occurrences) that do not seem casual, such as in the recently demonstrated interplay between BMF and immune deregulation [34].

Finding novel variants in a known gene, especially if classified with an uncertain significance, may require additional investigations to prove their association with specific phenotypic patterns [28], that is, more than mere in silico predictions [48,49]. The effect of some variants of the *CASP10* and *PIK3CD* genes, found in patients showing symptoms and laboratory alterations similar to ALPS patients (the so-called ALPS undefined, or ALPS-U), but not fully matching the 2009 NIH revised diagnostic criteria [11], was investigated through proper functional tests, allowing confirmation of their postulated pathogenicity [34,44,45].

Our attention was also attracted by a number of other genes whose variants, despite apparently not being correlated with disease phenotypes as either benign, likely benign, or of uncertain significance, may have had an impact in the respective conditions, such as the case of the remarkably high frequency of heterozygous *RAG1* variants (8/149 = 5.37%), affecting either the zinc binding domain (Zn-BD) (p.Asp887Asn; p.Asn968Lys; p.Ser982Tyr) or the nonamer binding domain (NBD) (p.Gln407Glu; p.Arg449Lys). In particular, the p.Asn968Lys variant is very close to the conserved catalytic amino acid p.Glu965, thus likely altering the structure of the catalytic domain and the DNA binding capability, and for this reason, it is reported as likely pathogenic (https://www.ncbi.nlm.nih.gov/clinvar/variation/36713/ (accessed on 7 January 2021)) [50]. Some of the variants we detected, despite being mono-allelic, may have a biological impact on the clinical phenotype, or, in the most evocative cases, undetected null alleles affecting noncoding or regulatory portions of the gene could account for the second allele in *RAG1*-associated recessive disorders [41,51].

The *TNFRSF13B* and *TNFRSF13C* genes also provided variants illustrating the often complicated and unclear genotype-phenotype correlations. Indeed, mutations of the former gene, also known as TACI, though rare, have already shown to vary between disease susceptibility and pathogenesis, with clinical presentation ranging from unaffected to severe immunodeficiency and also occurring in healthy controls [49,52,53]. Nonetheless, asymptomatic family members have been reported with detectable in vitro B cell defects, thus suggesting that the penetrance of some mutations could be higher in cells than for the clinical phenotype [54]. The *TNFRSF13B* gene is also present in our dataset with heterozygous variants, such as p.Ala181Glu, known to represent risk factors not enabling a genetic diagnosis [40,55].

On the other hand, the p.His159Tyr variant of the *TNFRSF13C* gene, though supposed to be benign, has a CADD score = 26.6, with two out of three software packages predicting a damaging effect, and it recurred in eight unrelated patients with a frequency in our dataset 3.6-fold higher than the frequency reported by GnomAD, a circumstance suggestive of a role, even marginal, in the disease manifestation.

Finally, we cannot rule out possible synergistic effects of multiple variants of different genes present in a number of patients reported in Table 1 and Table 2. This is the case of ID35, 86, 88, 90, 100, and 203, where pathogenic variants, likely pathogenic variants, or variants of unknown significance and a probable effect on the phenotype of two different genes might account, either alone or in a digenic mode of transmission, for the corresponding IEI disorders. Unfortunately, with the exception of ID100 whose two variants were both inherited from her father, thus excluding a digenic transmission, parental pairs for all the other patients were unavailable to prove inheritance from both parents.

Among the unsolved patients, namely, those left with no genetic diagnosis, we cannot exclude the possibility of novel genetic/clinical entities, especially in the light of the many atypical cases included in our cohort. Indeed, novel genetic causes of IEI are likely to be enriched in negative cases that can also include (1) defects in genes not included in our panel because they are not yet described in the literature, even in the case of the use of the CES [23], (2) defects located in regulatory regions not sequenced by targeted panels, and (3) missed detection of copy number variants (CNVs) and regions of homozygosity [56,57]. This limitation of our study might indeed account for a proportion of those cases that are heterozygotes for variants of genes responsible for recessive conditions, with undetected large indels affecting the second apparently normal allele. Given the suitability of unbiased approaches for broader genomic analysis, whole-exome sequencing or whole-genome sequencing may become a second-tier approach in IEI and autoinflammatory diseases to achieve a molecular diagnosis, especially in complex cases presenting atypical phenotypes or combinations of inflammatory phenotypes with immune defects.

## 5. Conclusions

The NGS approach applied to IEI demonstrated performances in line with the expectations. However, due to the remarkably variable clinical presentations and genetic diversity, defining the appropriate list of genes to design these panels may not be straightforward. In our experience, given a heterogeneous patient set, the best resolution was obtained using the widest panels, a result obviously expected and an observation testifying in favor of the application of unbiased approaches, especially when patients show atypical clinical pictures. Finally, focusing on the functional study of the many emerging variants, especially those of uncertain significance, will become an urgent need to reconcile inconsistent correlations between genotypes and clinical findings.

## Figures and Tables

**Table 1:** Pathogenic and likely pathogenic variants detected among 272 patients affected with inborn errors of immunity.

ID	GENDER	Total Variants Called	Filtered Variants ‡	Gene	Inherit. of Associated Phenotype	Variants §	ClinVar	Zygosity	Variant Classific. *	dbSNP (#rs)	CADD Score	Frequency (gnomAD)	DANN Score	FATHMM	SIFT	PROVEAN	Cases with Same Variant (#)	Parental Inheritance/*de novo*	Previous Genetic Tests	Clinical Phenotype	Consensus ***
6	F	592	17	*TNFRSF13B*	AD/AR	p.C104R	conflicting	HET	P	34557412	25.8	3.92 × 10^−3^	0.9172	D	D	D	4	M		ALPS-like	1
10	F	1335	21	*C9*	-	p.C125 *	-	HET	LP	na	32	-	0.985	-	-	-	1	M		ALPS-like	4
15	M	516	16	*RAG1*	AR	p.R507Q	-	HOMO	LP	143969029	28.7	6.57 × 10^−6^	0.9994	T	D	D	1	M, F		ALPS-like	1
19	F	497	14	*IKBKG*	XLR	p.E125K	B/LB	HET	LP	148695964	28	1.50 × 10^−3^	0.9991	D	D	D	2	M WT F na		ALPS-like	2
22	M	528	12	*TNFRSF13B*	AD/AR	p.C193 *	conflicting	HET	LP	72553885	35	5.68 × 10^−5^	0.985	-	-	-	2	F		ALPS-like	1
32	F	594	11	*STAT3*	AD	p.K658R	LP	HET	LP	na	25.5	-	0.999	D	T	N	1	Somatic		ALPS-like	1
				*ADA2*	AR	p.L188P	uncertain significance	HET	LP	760102576	26.8	1.97 × 10^−5^	0.999	D	D	D	1	M			1
				*ADA2*	AR	p.T187P	-	HET	LP	752890414	26.3	3.99 × 10^−6^	0.996	T	D	D	1	F			
35	F	640	13	*CTLA4*	AD	p.C58S fs*13	P	HET	LP	na	-	-	0.991	-	-	-	1	M	Sanger *FAS*	ALPS-like	1
39	M	661	8	*LRBA*	AR	p.R655 *	-	HOMO	P	199750191	42	6.58 × 10^−6^	0.998	-	-	-	1	M, F		ALPS-like	1
51	F	655	11	*ELANE*	AD	c.597 +1G>A	P	HET	P	1555710005	26.7	-	0.907	-	-	-	1	na		Neutropenia	1
64	F	1928	33	*RPS19*	AD	p.R62W	P	HET	P	104894711	24.9	-	0.999	D	-	-	1	M		BMF	1
66	M	683	10	*SMARCAL1*	AR	p.R499W	-	HET	LP	1302790588	25.3	3.98 × 10^−6^	0.9987	D	D	D	1	na		ALPS-like	4
75	F	718	12	*RAG1*	AR	p.Q407E	LP	HET	LP	na	25.1	-	0.986	T	D	N	1	M	Sanger *ELANE*	ALPS-like	2
80	M	1225	14	*IKBKG*	XLR	p.E125K	B/LB	HEMIZIG	LP	148695964	28	1.50 × 10^−3^	0.9991	D	D	D	2	M		ALPS-like	1
86	M	1470	21	*C8B*	AR	p.R428 *	P	HET	P	41286844	41	3.98 × 10^−6^	0.9984	-	-	-	1	na		BMF	4
				*FAN1*	AR	p.M86G fs*14	-	HET	LP	758406790	-	1.19 × 10^−5^	-	-	-	-	1	na			2
88	F	1386	22	*TNFRSF13B*	AD/AR	p.C104Y	LP	HET	LP	72553879	24.7	1.58 × 10^−4^	0.7764	D	D	D	2	na		ALPS-like	1
90	M	1105	16	*NHEJ1*	-	p.R57 *	P	HET	P	118204451	37	7.95 × 10^−6^	0.997	-	-	-	1	na		ALPS-like	1
92	M	1196	18	*C7*	-	R521S	P	HET	LP	121964920	22.3	2.35 × 10^−3^	0.9973	T	D	D	1	F		ALPS-like	1
93	M	131	3	*TNFRSF13B*	AD/AR	p.C193 *	conflicting	HET	LP	72553885	36	3.99 × 10^−6^	0.985	-	-	-	2	na		ALPS-like	1
94	F	1292	22	*NCF1*	AR	p.W193 *	P	HOMO	P	145360423	36	5.53 × 10^−4^	0.995	D	-	-	1	M, F		Immune-deficiency	1
97	M	815	28	*IL7R*	AR	p.C118Y	P	HOMO	LP	193922641	19.9	3.95 × 10^−5^	0.9369	T	T	D	1	na	Sanger *TERC, TERT*	Immune-deficiency	1
100	F	1614	22	*AIRE*	AR	p.E517 *	-	HET	P	na	48	-	0.994	-	-	-	1	F		ALPS-like	4
105	F	1517	26	*AIRE*	AR	p.R9W	LP	HET	LP	na	23.6	-	0.998	D	D	D	1	na	Sanger *FAS*	ALPS-like	4
106	F	703	25	*RNASEH2B*	AR	p.A177T	P/LP	HET	P	75184679	24	1.45 × 10^−3^	0.9967	D	T	N	1	na		BMF	2
109	F	1603	26	*TMEM173*	AD	p.V155M	P	HET	P	587777610	24.7	2.63 × 10^−5^	0.999	T	D	N	1	na		SAID	1
113	F	1780	35	*CASP8*	AR	p.R494 *	-	HET	P	1368296717	37	3.98 × 10^−6^	0.996	-	-	-	1	na	Sanger *TERC*	Immune-deficiency	2
114	F	1123	10	*TNFRSF13B*	AD/AR	p.L69T fs*12	conflicting	HET	LP	72553875	22.8	3.09 × 10^−4^	-	-	-	-	3	na		ALPS-like	1
120	M	1736	33	*TNFRSF13B*	AD/AR	p.S194 *	P	HET	P	121908379	36	-	-	-	-	-	1	na		ALPS-like	1
131	M	1450	25	*STAT3*	AD	p.R152W	P	HET	LP	869312890	25.7	0.00	0.998	T	D	D	1	na	Sanger *FAS*	ALPS-like	1
135	M	1095	28	*SH3BP2*	AD	p.T531I	-	HET	LP	746860671	21.7	3.98 × 10^−6^	0.9927	T	D	N	1	na		ALPS	3
139	M	174	11	*SBDS*	AR	c.258+2T>C	P	HET	P	113993993	33	3.88 × 10^−3^	-	-	-	-	2	na	Sanger *FAS, ADA2*	ALPS-like	2
162	M	106	4	*TNFRSF13B*	AD/AR	p.C104R	conflicting	HET	P	34557412	25.8	3.92 × 10^−3^	0.9172	D	D	D	4	na	Sanger *TERC, TINF2*	BMF	1
178	M	111	5	*SBDS*	AR	c.25 +2T>C	P	HET	P	113993993	33	3.88 × 10^−3^	-	-	-	-	2	na		Hystocytosis	4
182	M	169	11	*RAB27A*	AR	p.I181M	uncertain significance	HET	LP	139025012	17.7	9.19 × 10^−5^	0.9953	T	D	N	1	na		AIHA	4
192	F	129	3	*FAS*	AD	c.650_651+3del CTGTA insAGTG	uncertain significance	HET	LP	na	14.95	3.98 × 10^−6^	0.8238	-	-	-	1	na		ALPS-like	1
203	F	140	6	*TNFRSF13B*	AD/AR	p.L69T fs*12	conflicting	HET	LP	72553875	22.8	3.09 × 10^−4^	-	-	-	-	3	na		ALPS	1
206	F	148	4	*SBDS*	AR	p.K62 *	P/LP	HET	P	120074160	45	1.67 × 10^−4^	0.996	-	-	-	1	na		BMF	2
209	M	144	4	*TNFRSF13B*	AD/AR	p.C172Y	uncertain significance	HET	LP	751216929	22.2	1.90 × 10^−4^	0.7465	D	D	D	1	na	Sanger *TERC*	ALPS	1
220	M	141	4	*TERT*	AD	p.E429 *	-	HET	LP	na	32	-	0.994	-	-	-	1	F	Sanger *TERC, TINF2*	BMF	1
226	F	122	6	*TNFRSF13B*	AD/AR	p.C104Y	LP	HET	LP	72553879	24.7	1.58 × 10^−4^	0.7764	D	D	D	2	na		Neutropenia	1
				*ELANE*	AD	p.P139L	P/LP	HET	P	137854448	23.6	-	0.999	D	D	D	2	na			1
242	M	127	2	*TINF2*	AD	p.R282C	P	HET	P	121918545	26.9	0.00	0.999	D	D	D	1	na		BMF	1
252	F	128	4	*TNFRSF13B*	AD/AR	p.L69T fs*12	conflicting	HET	LP	72553875	22.8	3.09 × 10^−4^	-	-	-	-	3	na		ALPS	1
253	M	154	12	*ELANE*	AD	p.P139L	P/LP	HET	P	137854448	23.6	-	0.999	D	D	D	2	na	Sanger *HAX1*	Neutropenia	1
1176	M	832	16	*MVK*	AR	p.L168_ D170 delinsHis	uncertain significance	HET	LP	na	-	-	-	-	-	-	1	na	Sanger *MVK, TNFRSF1A*	ALPS-like	1
						p.V377I	conflicting	HET	P **	28934897	15.11	1.47 × 10^−3^	0.981	D	T	N	1	na			
2130	F	1834	16	*NOD2*	AD	p.W709 *	-	HET	LP	776701942	36	8.03 × 10^−6^	0.985	-	-	-	1	F	PMID: 26386126	SAID	1
260	F	118	4	*CARD11*	AD	p.M1I	uncertain significance	HET	P	na	22.4	-	-	T	D	-	1	na		Neutropenia	1
288	F	122	6	*LRBA*	AR	p.Q2717 *	-	HET	P	na	50	-	-	-	-	-	1	na		ALPS-like	1
						p.E946 *	-	HET	LP	777413769	24.2	3.94 × 10^−5^	-	-	-	-	1	na			
285	M	132	5	*TNFRSF13B*	AD/AR	p.I87N	conflicting	HET	LP	72553877	24.6	3.48 × 10^−4^	-	D	D	-	1	na		ALPS-like	1
303	M	110	4	*FAS*	AD	p.H282R fs*14	-	HET	LP	na	-	-	-	-	-	-	1	na		ALPS-like	1
307	M	117	4	*TNFRSF13B*	AD/AR	p.C104R	conflicting	HET	P	34557412	25.8	3.92 × 10^−3^	0.9172	D	D	D	4	na		ALPS-like	1
313	M	138	3	*FAS*	AD	p.Gly66C	-	HET	P	na	34	-	-	D	D	-	1	na		ALPS-like	1
316	M	147	7	*TNFRSF13B*	AD/AR	p.C104R	conflicting	HET	P	34557412	25.8	3.92 × 10^−3^	0.9172	D	D	D	4	na		ALPS-like	1

Blank lines report heterozygotes for variants that, though predicted with a causative effect, are either responsible for recessively inherited phenotypes or inconsistent with the clinical phenotype. Grey lines report patients with causative variants of genes compatible with their clinics, showing a zygosity consistent with the inheritance mode of the disease. T = tolerated; D = damaging; N = neutral. ‡ variants filtered according to location: exonic and splicesite ±5; function: missense, frameshift, stoploss, stopgain; frequency: MAF and EMAF ≤ 0.05. § only validated (true positive) variants are reported; variants that could not be validated (false positive) and variants not followed up (considered not to contribute to the phenotype) are not reported. Parental segregation: F = father; M = mother; na = not available. * variant classification is according to ACMG criteria as reported in the Varsome website (https://varsome.com/ (accessed on 14 January 2021)). ** classification is according to INFEVERS database (https://infevers.umai-montpellier.fr/web/search.php? (accessed on 14 January 2021) *n* = 3). *** CONSENSUS: 1. gene associated with the patient’s pathology + zygosity consistent with heredity + variant classified P/LP on Varsome. 2. gene associated with the patient’s pathology + zygosity NOT consistent with heredity + variant classified P/LP on Varsome. 3. gene NOT associated with the patient’s pathology + zygosity consistent with heredity + variant classified P/LP on Varsome. 4. gene NOT associated with the patient’s pathology + zygosity NOT consistent with heredity + variant classified P/LP on Varsome. ALPS = Autoimmune lymphoproliferative syndrome; SAID = Systemic AutoInflammatory Disorder; AIHA = Autoimmune hemolytic anemia; BMF = Bone Marrow Failure. DANN, FATHMM, SIFT and PROVEAN scores have been deduced by the Varsome website. CADD score was obtained from https://cadd.gs.washington.edu/ (accessed on 14 January 2021).

**Table 2:** Variants of unknown significance with a probable effect on the phenotype, detected among 272 patients affected with inborn errors of immunity.

ID	GENDER	Total Variants Called	Filtered Variants ‡	Gene	Inherit. of Associated Phenotype	Variant §	CLINVAR	Zygosity	Variant Classific. *	dbSNP (#rs)	CADD Score	Frequency (gnomAD)	DANN Score	FATHMM	SIFT	PROVEAN	Cases with Same Variant	Parental Inherit./*de novo*	Previous Genetic Tests	Clinical Phenotype
2	F	631	11	*PRKCD*	AR	p.G248S	-	HET	VUS	144320413	28.9	6.57 × ^10−6^	0.9989	D	D	D	1	*de novo*		ALPS-like
14	F	571	15	*RAC2*	AD	p.R68Q	-	HET	VUS	na	29.7	-	0.9996	T	D	D	1	na		ALPS-like
29	M	716	11	*WRAP53*	AR	p.G481S	-	HET	VUS	763828661	26.6	6.58 × 10^−6^	0.9985	D	D	D	1	na	Sanger *TERC, TERT*	BMF
35	F	640	13	*LRBA*	AR	p.D2294N	-	HET	VUS	939898061	26.3	7.97 × 10^−6^	0.999	T	D	D	1	M	Sanger *FAS*	ALPS-like
40	F	534	13	*CARD11*	AD	p.R967C	uncertain significance	HET	VUS	149857605	24.8	5.26 × 10^−5^	0.9988	T	D	D	1	M		ALPS-like
48	M	594	11	*LYST*	AR	p.R2624W	conflicting	HET	VUS	150306354	26.3	2.81 × 10^−3^	0.9991	T	D	D	1	M		Neutropenia
71	F	1762	28	*RAG2*	AR	p.G509D	-	HET	VUS	779267024	15.52	7.97 × 10^−6^	0.9969	D	D	N	1	na	Sanger *TERC, TERT, TINF2, DKC1*	ALPS-like
73	F	996	18	*WDR1*	AR	p.T478M	-	HET	VUS	186889066	25.5	7.68 × 10^−4^	0.9931	T	D	D	1	na		ALPS-like
86	M	1470	21	*ATM*	AR	p.R2912G	-	HET	VUS	376676328	26.2	2.04 × 10^−4^	0.9986	D	D	D	1	na		BMF
87	M	1827	21	*AIRE*	AR	p.R356W	-	HET	VUS	376901046	22.3	1.45 × 10^−4^	0.9979	D	D	D	1	na		BMF
				*BLNK*	AR	p.G30R	-	HET	VUS	143109144	25.4	7.18 × 10^−4^	0.9993	-	D	D	1	na		
88	F	1386	22	*ATM*	AR	p.Y67C	uncertain significance	HET	VUS	754033733	25.6	4.02 × 10^−6^	0.9975	T	D	D	1	na		ALPS-like
90	M	1105	16	*CXCR4*	AD	p.L125V	-	HET	VUS	1001278766	26.2	1.31 × 10^−5^	0.9974	T	D	D	1	na		ALPS-like
100	F	1614	22	*TNFRSF13B*	AD/AR	p.E117Gfs*35	uncertain significance	HET	VUS	na	-	-	-	-	-	-	1	F		ALPS-like
102	M	1961	34	*RAG2*	AR	p.L279P	uncertain significance	HET	VUS	na	26.7	-	0.9985	D	D	N	1	na		ALPS-like
103	M	1509	39	*FANCA*	AR	p.A430V	uncertain significance	HET	VUS	772567344	22.4	6.57 × 10^−6^	0.9947	D	T	D	1	na	Sanger *TERC*	ALPS-like
110	F	1349	16	*NLRC4*	AD	p.R492W	uncertain significance	HET	VUS	1317272776	22.3	3.98 × 10^−6^	0.9787	T	D	D	1	na		ALPS-like
				*STAT5B*	AD/AR	p.R100C	uncertain significance	HET	VUS	199894785	32	7.24 × 10^−5^	0.9994	T	D	D	1	na		
124	M	1443	19	*CHD7*	AD	p.S1406R	LP	HET	VUS	na	22.3	-	0.995	T	T	D	1	F		BMF
132	F	1607	23	*C1S*	AD	p.R534W	uncertain significance	HET	VUS	121909582	26.8	2.10 × 10^−4^	0.9992	D	D	D	1	na		ALPS-like
159	M	134	3	*G6PC*	AR	p.T267M	-	HET	VUS	145296477	21.6	7.56 × 10^−5^	0.998	T	T	N	1	na		ALPS-like
172	M	133	4	*LRBA*	AR	p.R2862C	conflicting	HET	VUS	145709687	27.5	1.47 × 10^−3^	0.9992	T	D	D	1	na		ALPS-like
174	M	134	6	*AP3B1*	AR	p.V315A	uncertain significance	HET	VUS	na	29.7	-	0.9986	T	D	D	1	na	Sanger *FAS*	ALPS-like
176	F	134	3	*WAS*	XLR	p.E131K	B/LB	HET	VUS	146220228	24.6	2.16 × 10^−3^	0.9991	D	D	D	1	na	Sanger *FAS*	ALPS-like
203	F	140	6	*TERT*	AD/AR	p.E441del	conflicting	HET	VUS	377639087	-	1.72 × 10^−3^	-	-	-	-	1	na		ALPS
204	M	140	6	*ITK*	AR	p.Y240C	uncertain significance	HET	VUS	na	27.4	-	0.9982	T	D	D	1	na		AIHA
205	M	155	6	*CTC1*	AR	p.P999H	uncertain significance	HET	VUS	780572571	16.72	3.19 × 10^−5^	0.9453	D	D	D	1	na	Sanger *TERT, TERC*	Neutropenia
214	M	131	4	*CARD11*	*AD/AR*	p.S439F	uncertain significance	HET	VUS	760856731	28.1	2.79 × 10^−5^	0.9979	T	D	D	1	na		Neutropenia
2130	F	1834	16	*MPL*	AD/AR	p.R537Q	-	HET	VUS	3820551	26	9.21 × 10^−5^	0.993	D	D	N	1	na	PMID: 26386126	SAID
2582	M	1703	34	*STXBP2*	-	p.I74F	-	HET	VUS	na	26.6	-	0.9899	D	D	D	1	na	PMID: 31325311	SAID
261	M	116	2	*CARD11*	AD/AR	p.V90F	-	HET	VUS	na	25.6	-	-	D	D	-	1	na		ALPS-like
301	M	107	9	*PIK3CD*	AD/AR	p.P864L	uncertain significance	HET	VUS	148984508	26	-	-	D	D	-	1	na		Neutropenia
315	F	129	3	*FAS*	AD	p.C135Y	uncertain significance	HET	VUS	na	25.5	-	-	D	D	-	1	na		ALPS-like

Blank lines report variants of unknown significance with a probable effect on the phenotype, unreported thus far in association with any disease phenotypes, being in fact very rare with pathogenicity scores predicting damaging effects. Gray lines report variants affecting genes consistent with the corresponding patient’s phenotype and with zygosity concordant with the inheritance mode of the disease. T = tolerated; D = damaging; N = neutral. ‡ Variants filtered according to location: exonic and splicesite ±5; function: missense, frameshift, stoploss, stop-gain; frequency: MAF and EMAF ≤ 0.05. § Only validated (true positive) variants are reported; variants that could not be validated (false positive) and variants not followed up (considered not to contribute to the phenotype) are not reported. * Variant classification according to ACMG criteria as reported on the Varsome website (https://varsome.com/ (accessed on 7 January 2021)). Parental segregation: F = father; M = mother; na = not available. ALPS = autoimmune lymphoproliferative syndrome; SAID = systemic autoinflammatory disorder; AIHA = autoimmune hemolytic anemia; BMF = bone marrow failure. DANN, FATHMM, SIFT, and PROVEAN scores were deduced by the Varsome website. CADD score was obtained from https://cadd.gs.washington.edu/ (accessed on 14 January 2021).

**Table 3:** Distribution of variants among patients (A) and among predicted effects (B).

**A_Variant Distribution among the 272 Patients Studied**			
*n* = 0	*n* = 1	*n* = 2	*n* ≥ 3
103	114	38	17
37.9%	41.9%	14%	6.2%
**B_Classification of the 197 Different Variants Detected**			
Pathogenic/Likely Pathogenic	VUS with a probable effect on the phenotype		VUS Low impact/Likely Benign/Benign
47	33		117

## Data Availability

Any data supporting the findings of this study are available on request from the corresponding author. Moreover, seventy-eight novel variants, reported here for the first time, have been submitted to ClinVar (https://www.ncbi.nlm.nih.gov/clinvar/ (accessed on 1 August 2021)) and assigned accession numbers SCV001424053-SCV001424130.

## Supplementary Materials

The following are available online at https://www.mdpi.com/article/10.3390/genes12091299/s1, Table S1: List of genes included in panels 1, 2, and 3, with the respective transcript RefSeq and coverage of the input design; Table S2: Technical details of gene panels 1, 2, and 3; Table S3: Run metrics of gene panels 1, 2, and 3; Table S4: Benign, likely benign, or low-impact variants of unknown significance detected among 272 patients affected by inborn errors of immunity.
